# How the evolutionary theory of aging can guide us in the search for aging genes

**DOI:** 10.18632/aging.100460

**Published:** 2012-06-06

**Authors:** Kevin Flurkey, Rong Yuan

**Affiliations:** The Jackson Laboratory, 600 Main Street, Bar Harbor, Maine 04609, USA

It is a stroke of irony that lifespan — the principal phenotype used to search for aging genes — is a terrible phenotype for genetic analysis. Lifespan has relatively low heritability under most conditions, and it is affected by chronic, age-related diseases that confound its use as a biomarker of aging. If the majority of aging genes are pleiotropic, as proposed by the evolutionary theory of aging, an opportunity is provided to identify these genes through the “back door,” using phenotypes that are more amenable to genetic analysis. To choose the pleiotropic phenotype for our studies, we went back more than 50 years to Williams [1], who, in his seminal paper, specified four “physiological expectations that follow from the theory,” two of which we applied: “Rapid individual development should be correlated with rapid senescence,” and, to specify a particular developmental phenotype, “The time of reproductive maturation should mark the onset of senescence.” Therefore, to search for genes that regulate lifespan, we looked in the other direction — for genes that govern reproductive maturation.

We started our search for pleiotropic aging genes by evaluating the age of female sexual maturity in the highly diverse, 32-inbred strain panel provided by The Jackson Laboratory Nathan Shock Center. As predicted by the evolutionary theory of aging, age of vaginal patency (VP) correlated positively with lifespan across strains. Interestingly, it also correlated negatively with circulating IGF1. And, because, in the same 32 strains of mice, 6-month IGF1 levels also correlate negatively with lifespan [2], it is likely that IGF1 mediates a significant portion of the linkage of reproductive maturation with aging and lifespan. We tested this idea in a congenic C57BL/6J (B6) mouse that carries a portion of Chr 10 from the C3H/HeJ strain. In congenic females, circulating IGF1 was elevated and as predicted, age of VP was accelerated and lifespan was shortened. (In males, IGF1 was not elevated and lifespan was not shortened.) Thus, the stage was set to begin a search for pleiotropic genes that influence both female reproductive maturation and lifespan in the strain panel.

Using haplotype association mapping, we identified three loci (*Vpq1* [Chr 4]*, Vpq2* and V*pq3* [both on Chr 16]) that accounted for 76% of the variance in age of VP across strains. Again, IGF1 may be involved. Age of VP was delayed and circulating IGF1 was diminished in the B6.PWD–Chr 16 consomic strain, in which the PWD/PhJ Chr 16, carrying the late age of VP haplotype at *Vpq3*, replaces the B6 Chr 16, carrying an intermediate age of VP haplotype. Haplotypes of *Vpq 3* also associated with differences in lifespan, strongly supporting the hypothesis that a gene at *Vpq3* mediates an antagonistic relationship between age of VP and lifespan.

We then used an arsenal of bioinformatic and genetic strategies to query *Vpq3* for candidate genes, which ultimately designated nuclear receptor interacting protein 1 (*Nrip1*) as a strong candidate. NRIP1 is a regulator of nuclear receptors that have broad functions in regulating cell growth, cell death, and metabolism. *Nrip1*, as a transcriptional repressor of PGC-1α and β, could promote senescence by suppressing mitochondrial biogenesis [3]. *Nrip1* null (−/−) mice are smaller and leaner, are resistant to high-fat diet-induced obesity, and show increased glucose tolerance and insulin sensitivity [4,5] — mimicking characteristics found in calorierestricted mice. We compared *Nrip1* null mice with their normal littermates. Results verified the hypothesis — generated by genetic and bioinformatic analyses — that knocking out *Nrip1* could significantly decrease IGF1 level and delay age of VP. Lifespan studies of *Nrip1* null mice are in process.

Additional work is necessary to verify *Nrip1* as an aging gene that mediates an antagonistic relationship between female reproductive maturation and lifespan. But our present analysis does provide direct support for the hypothesis that genes that regulate IGF1 constitute an entire category of pleiotropic genes that influence both reproductive maturation and aging. Our results also suggest that genes such as *Nrip1* — with highly pleiotropic effects on metabolism and cell turnover as well as on cell signaling and life history traits — are the types of genes that have the greatest impact on aging. Our work demonstrates that the utilization of pleiotropy in strategies informed by evolutionary theory is an effective approach in the search for genes that regulate aging.
